# Recent Advances in Chemotherapeutics for Leishmaniasis: Importance of the Cellular Biochemistry of the Parasite and Its Molecular Interaction with the Host

**DOI:** 10.3390/pathogens12050706

**Published:** 2023-05-12

**Authors:** Ranjeet Singh, Mohammad Kashif, Prateek Srivastava, Partha Pratim Manna

**Affiliations:** 1 Immunobiology Laboratory, Department of Zoology, Institute of Science, Banaras Hindu University, Varanasi 221005, India; ranjeet.singh10@bhu.ac.in (R.S.); kashifjmi.bioinfo@gmail.com (M.K.); prateekbhu.chem77@gmail.com (P.S.); 2 School of Computational and Integrative Sciences, Jawaharlal Nehru University, New Delhi 110067, India; 3 Department of Clinical Biochemistry and Pharmacology, Faculty of Health Sciences, Ben-Gurion University of the Negev, Beer-Sheva 84105, Israel

**Keywords:** chemoinformatics, bioinformatics, drugs, inhibitors, drug resistance, PPDK, ascorbate peroxidase, calcium ion

## Abstract

Leishmaniasis, a category 1 neglected protozoan disease caused by a kinetoplastid pathogen called *Leishmania,* is transmitted through dipteran insect vectors (phlebotomine, sand flies) in three main clinical forms: fatal visceral leishmaniasis, self-healing cutaneous leishmaniasis, and mucocutaneous leishmaniasis. Generic pentavalent antimonials have long been the drug of choice against leishmaniasis; however, their success is plagued with limitations such as drug resistance and severe side effects, which makes them redundant as frontline therapy for endemic visceral leishmaniasis. Alternative therapeutic regimens based on amphotericin B, miltefosine, and paromomycin have also been approved. Due to the unavailability of human vaccines, first-line chemotherapies such as pentavalent antimonials, pentamidine, and amphotericin B are the only options to treat infected individuals. The higher toxicity, adverse effects, and perceived cost of these pharmaceutics, coupled with the emergence of parasite resistance and disease relapse, makes it urgent to identify new, rationalized drug targets for the improvement in disease management and palliative care for patients. This has become an emergent need and more relevant due to the lack of information on validated molecular resistance markers for the monitoring and surveillance of changes in drug sensitivity and resistance. The present study reviewed the recent advances in chemotherapeutic regimens by targeting novel drugs using several strategies including bioinformatics to gain new insight into leishmaniasis. *Leishmania* has unique enzymes and biochemical pathways that are distinct from those of its mammalian hosts. In light of the limited number of available antileishmanial drugs, the identification of novel drug targets and studying the molecular and cellular aspects of these drugs in the parasite and its host is critical to design specific inhibitors targeting and controlling the parasite. The biochemical characterization of unique *Leishmania*-specific enzymes can be used as tools to read through possible drug targets. In this review, we discuss relevant metabolic pathways and novel drugs that are unique, essential, and linked to the survival of the parasite based on bioinformatics and cellular and biochemical analyses.

## 1. Introduction

Leishmaniasis is a group of vector-borne infectious protozoan diseases endemic to nearly one hundred countries [1]. Leishmaniasis is considered by the WHO to be a neglected tropical disease and a major international health challenge. In addition to malaria, it is the deadliest parasitic disease worldwide. Nearly 0.71–1 million new cases and approximately 20,000 to 65,000 deaths are reported annually, predominantly in socioeconomically vulnerable communities with limited access to essential medicines. Over 20 different species of *Leishmania* are reported to be infective to humans, categorized as Old World (Mediterranean countries, Asia, and Africa) and New World (America) forms. The dipteran fly *Phlebotomus* and its subspecies in the Old World and *Lutzomyia* in the New World are proven vectors for human leishmaniasis. The disease globally affects approximately 14 million people, with over one billion people at high risk of infection [2]. A World Health Organization (WHO)-sponsored epidemiological report indicates that there are nearly 12 million active cases of leishmaniasis. The incidence of cutaneous leishmaniasis is two to three times more common than visceral leishmaniasis [3,4]. At present, this disease results in 20,000 to 65,000 deaths reported annually and is included among the 18 most neglected tropical diseases (NTDs). Approximately one hundred species of these dipteran insects belonging to the genera *Phlebotomus* and *Lutzomyia* are known as the main vectors involved in biological transmission [4]. *Leishmania* has a complex life cycle characterized by the presence of digenetic stages: flagellated promastigotes and flagellated amastigotes [5]. The metacyclic promastigote form in sand flies is responsible for infection in healthy individuals. The amastigote form is known for its pathogenesis, having a spherical shape with a rudimentary flagellum. Amastigotes reside, propagate, and persist within the host’s mononuclear phagocytic cells [6]. Leishmaniasis represents a wide spectrum pathology ranging from less severe and self-curable cutaneous leishmaniasis (CL) to more severe and fatal visceral leishmaniasis (VL). The clinical symptoms due to parasitic infections are classified into three types of disease: cutaneous, mucocutaneous, and visceral leishmaniasis [5,6]. Cutaneous leishmaniasis is manifested by the development of skin lesions and is the most common type prevalent in the Middle East. Visceral leishmaniasis, on the other hand, is distinguished by the occurrence of hepatosplenomegaly, fever, and weight loss, and is considered as a serious health hazard for the infected individual. Mucocutaneous leishmaniasis (MCL) is characterized by damage to oral mucous membranes in the nose, mouth, and throat, which potentiates inflammation and face disfiguration [7]. Recently, a new subgenus, *Mundinia,* has been reported, and a member of this group (*L. martiniquensis*) causes VL in Southeast Asian regions. *L. martiniquensis* typically causes VL in humans and can be treated with amphotericin B as a first-line chemotherapeutic option. It has been reported that VL caused by *L. martiniquensis* has a higher relapse rate and occurs in individuals with HIV infection [8,9].

In addition to two other kinetoplastid pathogens, viz. for *Trypanosoma cruzi* and *Trypanosoma brucei*, the management of leishmaniasis requires integrated and multidisciplinary strategies that include vector control, enhanced diagnostics, and increased awareness of new therapies with safe and efficient medicines [10]. There is still no effective vaccine available, and the control of the disease primarily rests on chemotherapy, the majority of which is costly and has a wide array of side effects [10,11]. Pentavalent antimonials (sodium stibogluconate, meglumine antimoniate or generic formulations) have been used as standard drugs in countries such as India and Nepal for over 60 years and remain the primary treatment options in many endemic regions despite widespread parasite resistance [12,13]. A single dose of the polyene antibiotic amphotericin B demonstrated a 95% efficiency against visceral leishmaniasis in India [14]. Intravenous administration of liposomal amphotericin B has become a standard treatment in many countries but remains expensive, even for single-course treatments [15,16]. Miltefosine, an alkyl-lysophospholipid analog, was initially developed as an anticancer compound and is considered as a first-line effective oral drug against Leishmania [17,18]. Miltefosine has been used successfully for the treatment of VL in India since 2002 and has been incorporated into the visceral leishmaniasis elimination program for the Indian subcontinent [19]. Despite some success, miltefosine administration registers considerable resistance, with relapse in nearly 20% of patients post-treatment [20]. Miltefosine was also found to be effective against cutaneous and mucocutaneous leishmaniasis in South America, with considerable differences in percent success in therapy programs [21,22,23]. Recently, the aminoglycoside paromomycin has been approved for the treatment of VL. The efficacy of paromomycin against VL patients has been demonstrated in phase III clinical trials in India [24,25]. However, paromomycin has also shown shortcomings in efficacy across geographical regions, as demonstrated by its less than satisfactory trial in Sudan compared to India [26,27]. It is known that the drugs used act via different cellular and molecular mechanisms, causing a variety of outcomes including the apoptosis of parasites, but are also widely associated with variable toxicity and setbacks in sought-after results. These difficulties are further complicated by the emergence of drug resistance against parasites across the globe that have persisted alongside conventional chemotherapy practiced in endemic areas. This significantly lowers the susceptibility to drugs and the emergence of difficult-to-treat resistant variants of the same species [28,29,30].

Many investigators have highlighted the need to discover new drug targets employing the knowledge of parasite biochemistry to develop revolutionary new drugs by using emerging technologies. Several natural and synthetic drugs as well as repurposed drugs have been screened and attempted against free parasites in clinical scenarios. Anti-leishmanial peptides are one such strategy that has recently gained in importance, particularly with active promotion strategies by pharmaceutical companies [31,32]. The commercialization of peptide-based drugs needs to rely heavily on their utility and clinical success with ease of synthesis, water solubility, sound biocompatibility, selectivity, versatility, tenability, and low toxicity [33]. Antimicrobial peptides (AMPs) are small molecules (<100 amino acids long) with positive charges and amphipathic specificities (hydrophobic and hydrophilic regions). AMPs act by affecting membranes by destabilization/disruption of phospholipids and induce cell death by increasing the permeability of the cell membrane and are less likely to be selective to resistant variants [34,35]. AMPs also pass through the membrane and interrupt or destabilize nucleic acid or protein synthesis and/or compromise enzyme (protease) functions or cell membrane synthesis. Thus, AMPs are an interesting candidate for effective therapeutic success against leishmaniasis [36]. The leishmanicidal effects of these peptides have been published in recent reports including the structural characteristics and inevitable challenges [37,38,39]. In the present work, we have compiled and analyzed the main advances and trends in drug development against *Leishmania* including contributions from our laboratory for the identification and experimental evaluation of future therapeutics.

## 2. Chemotherapy in Leishmaniasis: Current Drugs, Limitations, and Challenges

**Figure 1:**
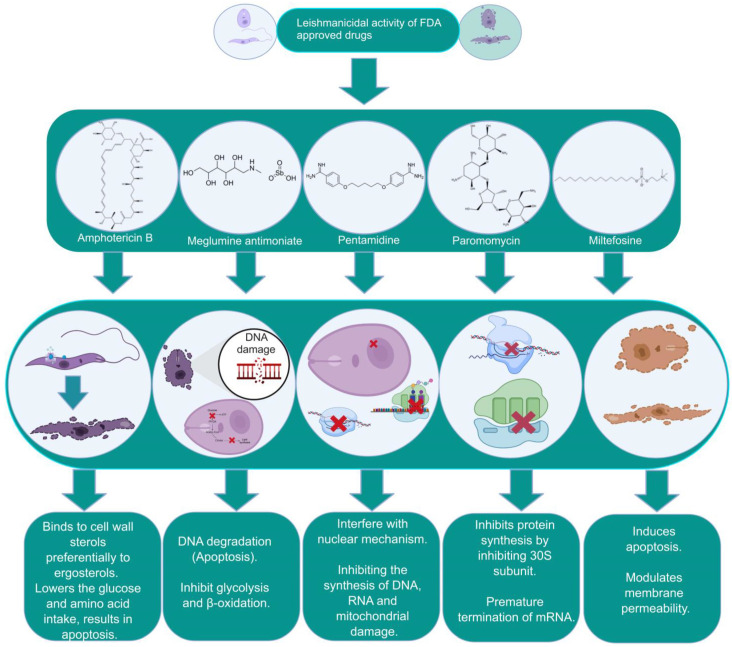
The biochemical characterization of FDA-approved drugs in Leishmania infection including the mode of action against the parasites.

The focus of this section relates to the discussion on the currently existing drugs in use for the treatment of VL. These include pentavalent antimonials, pentamidine, various formulations of amphotericin B (AmB), paromomycin, and miltefosine (Table 1 and Figure 1). These medications are also in use for the treatment of CL and MCL and PKDL. Treatment of VL considerably varies between the endemic regions spanning from India to Africa. The WHO approved and recommended regimens for known endemic VL foci are summarized in Table 1. Approximately 25 drugs or combinations are in use for humans with leishmaniasis [40,41,42].

WHO-OMS (2004) declared that liposomal amphotericin B, miltefosine, and paromomycin are the most promising drugs for the treatment of leishmanial infections. The search for potential new drugs and targets has been a very active area of research in the last couple of decades, with the publication of several important reviews [43,44,45,46,47,48,49,50,51,52,53,54,55,56].

**Table 1:** Details of the FDA-approved drugs for leishmaniasis, summarizing the cellular and molecular targets and limitations including side effects.

Drugs	Structure	Comments	Efficacy	Resistance	Uses	Toxicity	Ref.
**Meglumine** **antimoniate**	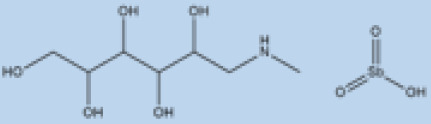	i.v. or i.m. First-line treatment.	Varies between 35 and 95% based on area.	High resistance in some regions of India.	VL, CL	Cardiotoxicity arthralgia, anorexia, fever, urticaria and significant toxicity to the liver, kidneys, and spleen. Hospitalization and constant monitoring of patients during treatment are needed.	[57,58]
**Paromomycin**	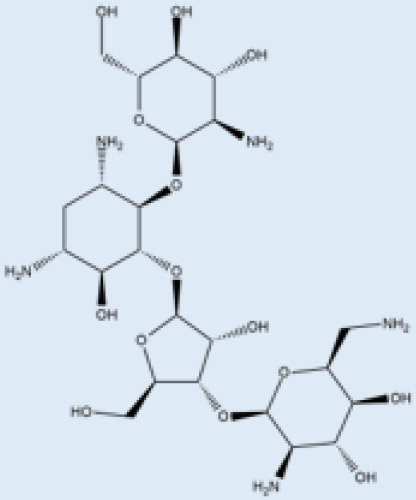	i.m.	A Phase III trial of Paromomycin (15 mg kg^−1^ (11 mg base) for 21 days showed 95% cure rate. Effective against PKDL.	No effective resistance.	CL, PKDL	Pain at the injection site, kidney toxicity, liver toxicity, and hearing toxicity.	[25,59]
**Amphotericin B**	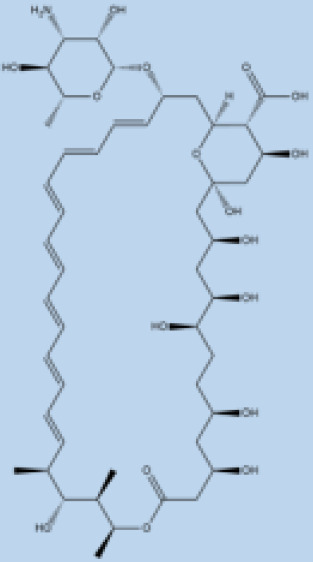	i.v. Very effective in regions with resistance.	>90%	No effective resistance.	VL	Infusion-related reactions, anemia, nephrotoxicity, myocarditis, and even death of the patient.	[14,60]
**Pentamidine**	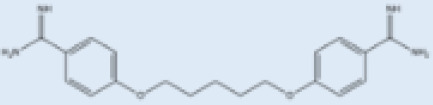	Pentamidine is a second-line leishmaniasis treatment that is mostly used for CL.	With cure rates ranging from 35% with *L. braziliensis* in Peru to 90% with *L. guyanensis* in Suriname, efficacy is very variable.	Yes	CL, VL	Heart damage, joint pain, loss of appetite, fever, urticaria, and serious liver, kidneys, and spleen damage. During treatment, patients must be hospitalized and constantly watched.	[55,61]
**Miltefosine**	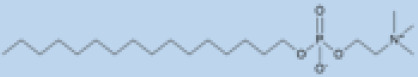	p.o. Teratogenic. Increasing treatment failures.	93–95% in India, 65–85% in Africa.	No effective resistance described.	CL, VL	It can cause birth defects, stomach problems, kidney damage, and liver damage and cannot be given to pregnant women.	[62,63]

A wide range of compounds of multiple families have been identified as potential hits and leads, and some of which are in clinical trials. Several candidates such as inhibitors impairing thiol metabolism, sterol, glycolytic, folate and trypanothione metabolism, etc. are important to consider (Table 2). These drugs and the commonly used chemotherapy (Table 1) still lack the ability to provide efficient control against *Leishmania*. Several combinations have been employed in clinical practice [11,64] including less toxic drug delivery systems (DDSs) such as PLGA nanoparticles or liposomes [55], poly-aggregated forms of AmpB [65], or amphiphilic antimony [66]. Below, we describe in more detail the current treatment options including their inadequacies and the need for new chemical entities.

### 2.1. Antimonials

Pentavalent antimonials (SbV) have been used in first-line chemotherapy based on parenteral administration available in the form of stibogluconate since 1945. It has been a sought-after monotherapy treatment for VL and is still in use against canine leishmaniasis [86,87]. Trivalent antimony (Sb^+3^) or emetic tartar was used against treatment for VL [57] but was later replaced by pentavalent antimony (Sb^+5^) compounds by Bramachari and called urea stibamine, which showed less toxicity than Sb^+3^ and emerged as an effective therapy against VL (kalazar) in India [88]. Pentavalent antimony is a prodrug that is converted to trivalent form to be active against the parasite. The interaction between host and intracellular amastigotes mediates the conversion [89,90]. The mechanism of action of the drug remains not completely understood and may include biochemical effects including the inhibition of DNA topoisomerase I, interference with the peculiar glutathione of trypanosomatids—trypanothione—and glycolytic enzymes [29,88,91,92]. The drug selectively accumulates intracellularly in parasites via modulation of the aqua glyceroporin AQP1 gene transporter [93,94,95], the overproduction of thiols, and the overexpression of ABC transporters (e.g., LABCI4, MRPA) [96,97,98]. Pentavalent antimony is available in two different formulations, Glucantime^®^ and Pentostam^®^, with an effectiveness of approximately 90% [99]. The main reasons for the restrictive use of antimonials include side effects/toxicity and the emergence of resistance and therapeutic failures in subcontinent countries. Patients receiving antimonials experience local pain with intramuscular injection and severe side effects including cardiotoxicity, pancreatitis, hepatotoxicity, and nephrotoxicity [58,100,101] (Figure 1).

### 2.2. Amphotericin

Amphotericin B (AmB) is a polyene antifungal obtained through the fermentation of *Streptomyces* nodosusparenteris used for treating leishmaniasis and acts via differential binding to ergosterol from Leishmania membranes [102,103]. AmB is effective against different species of *Leishmania* and is also recommended for pregnant women and patients who are coinfected with human immune deficiency virus (HIV). The success rate of AmB is above 90%; however, similar to pentavalent antimony, it also presents high toxicity in addition to its high cost [60]. The absence of ergosterol in mammalian cells makes its leishmanicidal activity important in clinical use without any significant incidence of drug resistance. Lipid formulations (liposomal) of amphotericin B viz. AmBisome^®^, Amphocil^®^, and Abelcet^®^ are less toxic than nonliposomal amphotericin B [104,105,106]. A phase III clinical trial with liposomal amphotericin in Bahia, Brazil for disseminated leishmaniasis, an emerging form of CL in the Americas, demonstrated a cure rate of 75% at doses >30 mg/kg [107]. Treatment with liposomal amphotericin B has fewer adverse effects and is considered more suitable for first-line treatment in Brazil [108]. In a murine model of VL, the polymeric micelle system and AmBisome^®^ caused significant scale-down in parasite load, inducing the generation of a pathogen-specific Th1 immune response without hepatic or renal damage. Treatment with amphotericin B deoxycholate and Glucantime^®^ caused significant toxicity to the infected animals [109] (Figure 1). Low-price liposomal AmpB (Fungisome^®^) and other drug delivery systems (e.g., microspheres of albumin, niosomes, chitosan, nanodisks, etc.) could be sustainable solutions for low-income regions of the world [110,111].

### 2.3. Miltefosine

Miltefosine, an alkyl phosphocholine derivative, was discovered as an antineoplastic agent for the treatment of cutaneous tumors, inducing apoptosis in tumor cells [112]. Miltefosine interferes with the cell membrane architecture by hindering phospholipid metabolism and affecting the synthesis of phosphatidylcholine and phosphatidylethanolamine by reducing intracellular choline [113]. The antileishmanial activity of miltefosine has been established in vivo and is now considered reliable chemotherapy against leishmaniasis with a clinical efficacy comparable to that of AmB [19,62,114]. Miltefosine is the only orally administered drug for treatment with an efficiency index of 95% in a clinical trial in India [62,63]. Miltefosine is also recommended in Ethiopia and South America [115]. The critical drawback of miltefosine administration is its long half-life (t1/2) in the organism (>120 h) plus its teratogenicity. This becomes a hindrance for its use in the fertile life of women. The efficacy of miltefosine in murine models varies depending on the type of *Leishmania* species. Susceptible BALB/c mice infected with *L. braziliensis* and *L. amazonensis* showed recurrence of the disease, which indicates a lack of efficiency of the drug in different types of leishmaniasis and may require very high doses for treatment [116,117,118]. Since miltefosine is taken orally, chances for the development of resistance due to self-medication habits without prescription are greater in countries such as India. The emergence of miltefosine resistance is relatively easy in *L. donovani* under laboratory conditions [119], and the loss of clinical efficacy has been reported [20,120,121] and confirmed in the laboratory [122] (Figure 1).

### 2.4. Pentamidine

Pentamidine is given intravenously or intramorally in patients who do not respond to pentavalent antimony [61]. Pentamidine showed high toxicity such as cardiotoxicity, reduction in blood pressure, and irreversible insulin-dependent diabetes mellitus [55] when used in VL. The drug binds to kinetoplastid DNA after its entry through arginine and polyamine transporters [55]. Additionally, the drug reported resistance, which has been associated with the upregulation of drug efflux, resulted in low levels of aromatic diamines in the cytosol and mitochondria [46] (Figure 1).

### 2.5. Paromomycin

Paromomycin (monomycin, aminosidine) is an aminoglycoside antibiotic obtained from *Streptomyces krestomuceticus* that acts by interfering with protein synthesis in the 16S ribosomal RNA ribosome of the target organism and inhibits respiration [123]. Paromomycin is effective against a variety of protozoan parasites (*Entamoeba*, *Giardia,* etc.) and its leishmanicidal activity has also been reported [124]. A phase III trial of paromomycin (15 mg kg^−1^ (11 mg base) for 21 days showed a 95% cure rate [25] and was approved in India for VL in 2006. Later, it was reported that the drug was ineffective in curing PKDL [59]. However, the application of paromomycin is plagued by the emergence of resistance when used in monotherapy despite its low cost and the absence of serious toxicity issues. Current chemotherapy for leishmaniasis has several limitations including high price, toxicity, onset of drug resistance, routes of administration, the length of treatment, and clinical failure. The emergence of severe forms of drug resistance to amphotericin B, miltefosine, etc. have increased alarmingly, particularly in endemic areas where the incidence of the outbreak is reported. It has been observed that the drugs are capable of resulting in clinical cure; however, infected individuals are not cured parasitologically, suggesting the remnants of the disease in the population. This has been reported in cutaneous and visceral *Leishmania* infection and is likely to be linked to the immunocompromised state of patients, where relapse is common in endemic areas in India and elsewhere. This condition highlights the impairment of the immune system due to parasite infection, suggesting the lack of effectiveness of the current drugs in inducing a long-term memory response to eradicate the disease (Table 1 and Figure 1).

## 3. Drug Resistance and Significance of Combination Therapy

Drug resistance is a phenomenon when the drug following selection showed reduced or no potential in its effect with reference to the susceptibility of a pathogen for a less than satisfactory effect. Clinical isolates of the pathogen have demonstrated natural variations in drug susceptibility, even in the absence of previous exposure to drugs. Reports from the Indian subcontinent have shown that parasite resistance originated mainly in areas of anthroponotic transmission [125]. Zoonotic transmission in the endemic region does not contribute to the prevalence of resistance, and only recently, unplanned urbanization may have changed this scenario, resulting in the emergence of drug-resistant parasites in those regions [126]. The incidence of HIV as a coinfection in leishmaniasis results in poor treatment outcomes with increased relapse rates and the emergence of potential drug resistance [127]. Sodium stibogluconate, meglumine antimoniate, or generic formulations have been the standard drug formulations for many decades in VL patients. In India and Nepal, the above drugs were rendered obsolete by 1995 due to drastic failure in the therapeutic efficiencies [128]. Clinical isolates of *L. donovani* from endemic regions have shown 3-fold less susceptibility in vitro than isolates derived from patients who respond to chemotherapy [12].

Drug resistance in *Leishmania* is due to the reduction in concentration of the drug in the parasite by decreased uptake mediated by the aquaglyceroporin AQP1, (the primary route of antimony entry) [129] or by the increased efflux of drug mediated by the ABC transporter ABCC3 (also known as MRPA) [96]. Antimony-resistant parasites also have increased levels of thiols (cysteine, trypanothione and glutathione) due to the overexpression/amplification of genes involved in the synthesis of glutathione and polyamines, the components of trypanothione, the main intracellular thiol in *Leishmania* [130,131,132,133,134]. Antimony resistance also occurs due to the inhibition of drug reduction or inactivation of the active drug [135]. Amphotericin B affects the membrane sterol of the parasite, ergosterol. Amphotericin B resistance is reported in 20% of Indian patients, where the drug was prescribed for VL patients refractory to antimonials [136]. Gene amplification in *Leishmania* alters the drug-binding affinity to the plasma membrane following modification in sterol composition [137]. Parasites derived from relapsing patients do not show differences in drug susceptibility in vitro [20], indicating that the reduced clinical efficacy is related to other factors such as the selection of parasites with increased virulence/infectivity, or inadequate interaction with the drug due to heterogeneous pharmacokinetics [138,139].

Miltefosine-treated parasites showed a significant reduction in mitochondrial membrane potential and cytochrome c oxidase activity [140]. Miltefosine binds to the plasma membrane and is internalized by the endocytic pathway via flippase activity mediated by the miltefosine transporter (MT) and its noncatalytic subunit Ros3. MT–Ros3 is responsible for the ATP-dependent accumulation of phosphocholine [117]. Miltefosine is excluded via exocytosis or by floppase activity, which may be mediated by the member subfamilies ABCB and ABCG of the ABC transporter [141].

In vitro susceptibility to miltefosine is intrinsically variable among the species type and clinical isolates of the pathogen [28,142]. Varying susceptibility among the different species and isolates may be due to fluctuations in the substrate specificity and activity of the MT–Ros3 machinery, rate of cell proliferation, biochemical targets, metabolism of the drug, and composition of the plasma membrane [143]. Recent studies suggest that the wide gap between in vitro susceptibility to miltefosine and treatment outcome in patients indicates an absence of correlation with the efficacy of the drug in the clinical setting [20,117]. In vitro miltefosine resistance is developed by increasing the concentration of the drug [142,144] or by chemical mutagenesis [145], likely associated with the defect in internalization of the drug, regulated by the MT–Ros3 axis. Following selection with miltefosine, MT and Ros3 genes underwent mutations, with MT genes showing a higher frequency of mutations [142,146,147,148]. MT inactivation induced a resistance phenotype in animal models of VL and CL, suggesting the importance of MT activity for the efficacy of miltefosine in vivo [142].

In CL patients, pentamidine has been used but showed toxicity when used in VL patients. Pentamidine-resistant lines obtained in vitro demonstrated alterations in the concentrations of intracellular arginine and polyamines, reduced pentamidine accumulation in the mitochondria, and augmented drug efflux [149], likely mediated by the ABC transporter PRP1 [150]. In addition, functional cloning using Cos-Seq identified a hypothetical protein that mediates the reduction in resistance to pentamidine by promastigotes [151].

Paromomycin causes alterations in the fluidity of the membrane, lipid metabolism, and mitochondrial activity. One paromomycin resistance gene has been identified in *Leishmania* encoding a hypothetical protein containing leucine-rich repeats conferring resistance to pentamidine [151]. Paromomycin susceptibility showed considerable heterogeneity in clinical isolates of different species of the parasite [152]. Incidence of treatment failure in leishmaniasis is a complex problem that may be caused by inappropriate handling of the drugs, patient-to-patient variability in susceptibility to the parasites, and the everlasting emergence of new isolates. In addition, pharmacokinetics and the immune response to individual drugs also play a pivotal role. The presence of *Leishmania* RNA virus 1 (LRV-1) in the Viannia subgenus poses an additional problem by subverting the host immune response by altering the effect of the drugs [153,154]. In light of the steady emergence of resistance against all antileishmanial drugs, a consensus on adopting combination therapy has been considered the preferred treatment option against fatal VL and others.

### Combination Therapy

Adopting a combination regimen will ideally reduce the dose of individual drug and the duration of the treatment and may bring about higher compliance and lower toxicity. The combination therapy in leishmaniasis may determine the best possible options and may assure its efficacy in clinical use for the available WHO-approved drugs used mainly by VL patients from the Indian subcontinent and Africa. Combination schemes include pentavalent antimonial plus paromomycin and AmB plus miltefosine. The first combination was tested in Africa by considering the widespread prevalence of antimonial resistance in Asia. Phase 2 and 3 trials in VL patients conducted in Bihar, India have assessed the combination of liposomal AmB (single dose) plus miltefosine administered for 7, 10, or 14 days [155]. A single-dose liposomal AmB and a 10-day course of intramuscular paromomycin or a combination of miltefosine and paromomycin for 10 days were tested in VL patients [156]. All of these blends were well-tolerated and demonstrated high cure rates of ~95% with a follow-up of 6–9 months. These combinations have advantages including reduced cost and time for therapy schedule, amount of administered drug, less toxicity, and the development of drug-resistant pathogens. VL patients coinfected with HIV have comorbidities, high fatality rates, and increased drug toxicities [157]. Protease inhibitors available against treatment for HIV patients have shown leishmanicidal activity in vitro [158]. A retrospective treatment strategy for HIV–*Leishmania* coinfected patients with liposomal amphotericin B plus miltefosine showed a positive outcome in the scheme [159]. This includes a lower relapse rate compared with monotherapy with liposomal AmB [160].

## 4. Structure- and Ligand-Based Drug Design: Antileishmanial Drug Discovery

### 4.1. Structure-Based Drug Design (SBDD)

**Figure 2:**
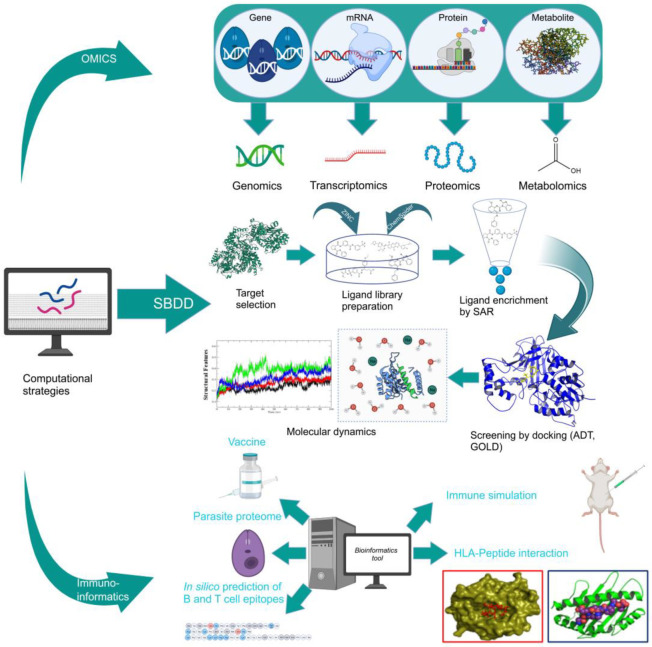
The application of various computational tools and strategies including OMICS, SBDD, and immunoinformatics for the rational design of novel therapeutics against Leishmania.

Combinatorial chemistry and high-throughput screening (HTS) have enabled the large-scale screening of compound libraries that include significant chemical diversity in a relatively short period [161,162]. According to sources, new medications on the market have decreased as a result of disappointing findings in various stages of clinical trials [163]. As a result, cost-effective methods for exploring and finding novel medications based on in silico or computational methods have evolved [164]. Protein structures generated by NMR or X-ray crystallography investigations are used in this method. In addition, a computationally based homology method can be used to model proteins using a variety of servers and tools. Then, for virtual screening of the protein active site pockets, small libraries of inhibitor molecules or lead compounds were produced. For this screening method, a number of docking tools are available, which aid in the discovery of the best hit compound by analyzing the compound’s binding affinity. According to Lipinski’s rule, compounds with a low binding affinity and no drug-like properties are discarded, while those with good interaction and high binding affinity are manufactured in laboratories by chemical vendors. These compounds have now been approved for use in biological systems [165]. SBDD has become a valuable tool for drug discovery and development in the field of medicine. Computational techniques and software could be used to improve the prediction of novel medications and synergistic pharmaceutical combinations to increase the treatment efficacy, avoid drug resistance, and lower dosage to avoid drug toxicity. There is some information and discussion regarding the SBDD strategy against trypanosomatides. The resources for studying leishmaniasis are listed in Table 3 and Figure 2. SBDD was utilized to find a pyrazolopyrimidine-class medication that was effective against *Leishmania* CDK12 (Cyclin-Dependent Kinase 12).

Lead compounds from the series proved appropriate and successful in a mouse model of infection when dosed orally twice a day for 10 days at 25 mg/kg, displaying equivalent efficacy to the front-line treatment miltefosine and decreasing parasite levels by 99% [177]. In one of the investigations, LASSBio-1386, an N-acylhydrazone derivative, was discovered to reduce the proliferation of *L. amazonensis* promastigotes while generating low cytotoxicity in macrophages. After in vitro treatment with LASSBio-1386, both the percentage of Leishmania-infected macrophages and the number of intracellular parasites were reduced. Furthermore, in vivo treatment of BALB/c mice infected with *L. amazonensis* resulted in a reduction in lesion size, parasite load, and histological architecture when compared to the controls. Molecular dynamics and docking studies were used to evaluate possible molecular interactions, and studies were performed on phosphodiesterase B1 of *Leishmania* (PDB code: 2R8Q) and LASSBio-1386. According to the computational research, LASSBio-1386 appears to work against *Leishmania* by altering leishmanial PDE (phosphodiesterase) activity [178]. Furthermore, new computational analyses are constantly being undertaken to find and propose new chemotherapy drugs [179,180,181,182].

This suggests that a computational method can assist in speeding up the development of novel anti-trypanosomatid drugs. This highly impactful approach potentiates the enhanced performance of the pharmaceutical industry in synthesizing better drugs across multifarious therapeutic horizons and has become a boon for increasingly complex disease management. Integration of computational tools into the research pipeline is an important innovation tool for finding new therapeutics. Chemoinformatics tools are classified as structure- and ligand-based drug design (SBDD and LBDD) approaches. SBDD methods use the 3D coordinates of molecular targets to study and optimize ligand–receptor interactions [183] and show the 3D architecture of numerous drug targets using X-ray crystallography. SBDD offers high affinity interactions with the targets by molecular docking, employing structure-based virtual screening (SBVS) where potential ligands are evaluated by virtue of their binding mode and energetics [184]. Structure–activity relationships (SAR) from these experiments could optimize the receptor–ligand affinity and other properties. Several macromolecular targets in *Leishmania* have been investigated for drug discovery. These include topoisomerases and proteases (cysteine proteases), tubulin, folate metabolism-related proteins, kinases, phosphodiesterases, and enzymes that participate in trypanothione and purine salvage pathways [185]. Ligands for these targets provide high-quality information for drug design.

Several other SBDD-based drug design attempts have been made to discover new targets against *Leishmania*. This includes a wide variety of pathways and biomolecules targeting the survival and physiological functions of parasites in host–parasite interactions. The list includes pteridine reductase 1 (PTR1), which is involved in the pteridine salvage pathway and folate metabolism [186]. Following molecular docking analysis using the crystal structure of *L. major* PTR1, active compounds were identified to have high-affinity interactions with the dihydropyrimidine and chalcone moieties of the enzyme catalytic site and leishmanicidal effects against promastigotes.

Cysteine proteases are recognized as another key enzyme responsible for parasite survival and the invasion of host cells [187]. Cathepsin-L-like endopeptidase CPB2.8 is a promising drug target in leishmaniasis. Benzimidazole derivatives displayed leishmanicidal potential against *L. infantum* amastigotes with binding potential to the catalytic site of CPB2.8 [188].

A novel quinalidine derivative has been identified as a suitable inhibitor against the mitochondrial enzyme NADH dehydrogenase (NDH2), which catalyzes electron transfer from NADH to ubiquinone and is another interesting candidate. Using homology modeling and pharmacophore-based virtual screening, novel NDH2 inhibitors in *L. infantum* have been screened for anti-leishmanial potential for in vivo studies in VL [189,190]. Quinalidine derivatives exhibit leishmanicidal activity in the nanomolar range against axenic cultures of both axenic amastigotes and promastigotes of *L. infantum*. SBVS of 53 leishmanial proteins including molecular dynamics simulations was performed for conformational structure following the screening of databases using the IBM World Community Grid [182]. An assembly of four proteins with high affinity interactions with the compounds in the database with the most favorable binding energy occurred in the *L. major* dihydroorotate dehydrogenase (LmDHODH) enzyme. LmDHODH mediates the oxidation of dihydroorotate in the pyrimidine synthesis pathway [191]. Ten top-scoring LmDHODH inhibitors were screened, selected, and assessed for in vitro leishmanicidal activity. Four of them were active against *L. panamensis* intracellular amastigotes, having leishmanicidal effects similar to that of the reference drug AmB. Furthermore, this drug did not show significant toxicity against human macrophages, indicating its potential for further development and future experimental studies including animal model studies.

Topoisomerase 1 of *L. donovani* (LdTop1) is another molecular target in the SBDD study [192]. Topoisomerase 1 causes single-strand breaks in DNA, enabling changes in topology, and is essential for cellular processes such as gene replication and transcription [193]. A series of LdTop1 inhibitors were identified by scaffold hopping and bioisosteric manipulations. Camptothecin and edotecarin are known Top1 inhibitors used as the standard starting inhibitors for constructing the molecular design. Six compounds were selected against LdTop1 by performing molecular docking studies using the crystal structures of LdTop1 and the human ortholog. Leishmanicidal activity was demonstrated against *L. donovani* promastigotes without any toxicity toward mammalian cells. The structure of the ternary complex 5-LdTop1-DNA, predicted by molecular docking analysis, revealed key structural aspects of the novel analogs with leishmanicidal activity without affecting the host cell cytotoxicity. Tryparedoxin peroxidase of *Leishmania* has been determined to be a suitable molecular target in SBDD, and the enzyme decreases hydroperoxides produced by infected macrophages. Thus, the enzyme is critically important for parasite survival [194]. Molecular docking analysis using the X-ray structure of the enzyme of *Leishmania major* (LmTXNPx) selected and designed a series of N,N disubstituted 3-aminomethyl quinolones with leishmanicidal properties that can be considered as suitable drug candidates against leishmaniasis.

### 4.2. Ligand-Based Drug Design

In cases where the X-ray 3D structure of the receptor is unavailable, a ligand-based design model is adopted to predict drug candidates. This methodology depends on information on the structure, molecular properties, and activity of the small molecules [195]. LBDD offers the construction of chemometric models that correlate molecular characteristics (molecular descriptors) with pharmacokinetic and pharmacodynamic parameters (target properties). Quantitative structure–activity and structure–property relationships (QSAR and QSPR, respectively) are derived to identify molecular characteristics that have a close relationship with the target property [196]. The LBDD protocol in combination with SBDD methods has been reported in drug discovery for leishmaniasis. LBDD uses QSAR and QSPR models for predicting activity and ADMET parameters and searches for novel compounds via ligand-based virtual screening (LBVS).

## 5. Design of Novel Drug Targets: Experience from our Laboratories

**Figure 3:**
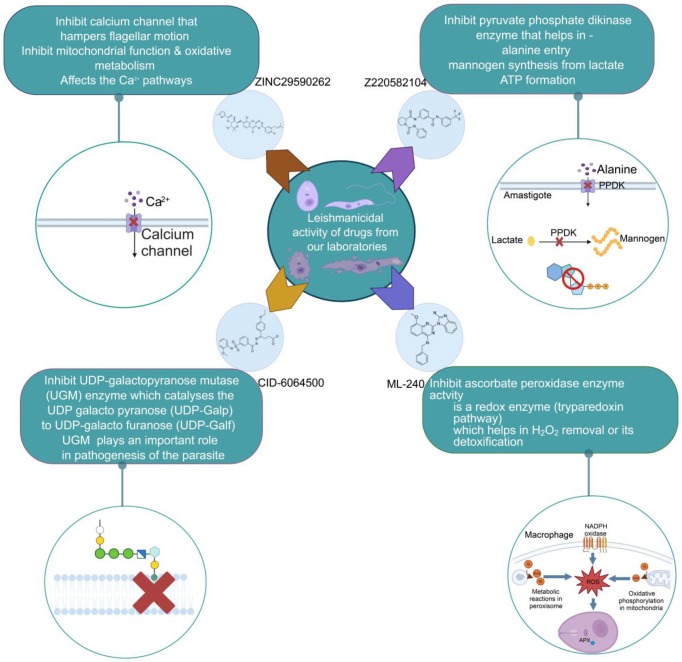
Depiction of the leishmanicidal potential of novel therapeutics targeted to the parasite’s specific survival pathways.

We studied four important cellular and biochemical pathways relevant to biochemical signaling events in *Leishmania*. These are associated with metabolic pathways that can be attractive targets for structure-based and ligand-based drug discovery approaches toward the development of novel antileishmanial therapeutics. We used the SBDD approach to identify putative inhibitors. In vitro and in vivo validations were performed to provide an effective platform for the evaluation of their efficiency for future antileishmanial drugs with minimal side effects. The major difficulties in SBDD application are the lack of tertiary or quaternary structures of many leishmanial proteins, which may play critical roles in numerous metabolic pathways related to housekeeping and parasite survival (Table 4 and Figure 3).

### 5.1. Pyruvate Phosphate Dikinase Inhibitor against Leishmania donovani

The identification of new and potentially effective inhibitors against the essential enzymes of parasites could offer new options for the treatment of VL [197]. 

Pyruvate phosphate dikinase (PPDK) is critical for the entry of alanine into intracellular amastigotes. The mammalian glucogenic precursor L-lactate is used by amastigotes during the synthesis of its storage carbohydrate mannogen in adverse conditions facilitated by PPDK [199]. PPDK catalyzes the reversible conversion of PPi, AMP, and phosphoenolpyruvate (PEP) into Pi, ATP, and pyruvate, respectively [200]. In mammals, the glycolytic pathway contains pyruvate kinase (PK) instead of PPDK for glucose synthesis. Thus, the absence of PPDK in humans and its indispensable role in *Leishmania* makes this enzyme an attractive target for antileishmanial drug design [201]. Drugs against *L. donovani* PPDK (*Ld*PPDK) could be economically affordable, have less chance to develop resistance, and deliver better antileishmanial effects. We identified a putative inhibitor with ID Z220582104 and compared it with miltefosine for antileishmanial activity against free promastigotes and intracellular amastigotes (Table 4). Z220582104 was found to be safe, tolerant, and nontoxic to mammalian cells, even at very high concentrations but significantly leishmanicidal against both forms of the pathogen. However, PPDK is less effective than miltefosine [179] (Figure 3). PPDK is also considered as a potential target for developing herbicides and a target enzyme for designing new drugs [202].

### 5.2. UDP-Galactopyranose Mutase of Leishmania Is a Drug Target

β-Galactofuranose (β-Galf) constitutes the cell surface matrix component of *Leishmania* and plays an important role in the pathogenesis of the parasite [203]. UDP-galactopyranose mutase (UGM) converts UDP-galactopyranose (UDP-Galp) to UDP-galactofuranose (UDP-Galf), which acts as a precursor for β-Galf synthesis. UGM is absent in humans; thus, the enzyme is a potential target for antileishmanial drugs [204]. The 3D protein structure of *L. major* UGM (*Lm*UGM) has been homology modeled by adopting *Trypanosoma* cruzi UGM (*Tc*UGM) as a template. Three compounds (6064500, 44570814, and 6158954) from the PubChem database among the top hits were selected that occupied the UDP binding site of *Lm*UGM, indicating a possible inhibitory role. In vitro antileishmanial activity was evaluated with the top ranked inhibitor 6064500 against promastigotes of *L. donovani*. Furthermore, at similar concentrations, the drug exhibited significantly higher levels of tolerance to mammalian cells than the standard drug miltefosine. Enamine (PubChem id: 6064500) showed concentration-dependent leishmanicidal activity against free promastigotes of *L. donovani* with an IC_50_ value equivalent to 50 μg/mL [197] (Figure 3).

### 5.3. Targeting Ascorbate Peroxidase of Leishmania

Oxidative stress is a host defense mechanism in macrophages that protects infected cells from pathogens by upregulating antioxidant moieties. Macrophages produce oxidative molecules (e.g., H_2_O_2_) that kill the parasites (*Leishmania donovani*), which in turn detoxifies the effects of H_2_O_2_ via a unique redox enzyme called ascorbate peroxidase (APX). Leishmania is deficient in catalase and glutathione (GSH) peroxidase, and thus H_2_O_2_ removal or detoxification is performed by the tryparedoxin pathway [205]. Overexpression of APX in *Leishmania major* (Lm-APX) protects against oxidative stress [206,207,208]. APX is important for parasite survival, and its absence in mammalian (human) hosts makes it an ideal target that could be used for therapeutic purposes. Blocking *L. donovani* APX (Ld-APX) in the ligand binding site by a novel inhibitor may alter the parasite’s oxidative stress potential for escape mechanisms. The crystal structure of Ld-APX is unavailable in the Research Collaboratory for Structural Bioinformatics (RCSB); thus, Ld-APX was modeled and screened using the ligand library prepared for the ascription of novel drug candidates.

Docking and MD simulation studies identified the inhibitor ZINC96021026, which is identical to the drug ML-240 that inhibits p97 ATPase activity with an approximate IC_50_ value of 100 nM [209]. ML-240 also inhibits p97-dependent degradation of proteasome substrates with an approximate IC_50_ of 900 nM [210]. AAA-ATPase p97 plays an important role in the protein homeostasis of eukaryotic cells by accentuating the degradation of ubiquitinated proteins by the proteasome and the maturation of autophagosomes [211,212]. ML-240 induces caspase 3 and 7 activation in SW403 and HCT15 cells and blocks tumor cell proliferation. ML-240 antagonizes p97 ATPase activity and was studied via high-throughput screening (HTS) of the NIH Molecular Libraries Small Molecule Repository (MLSMR) database. Our in silico observations indicated that ML-240 inhibits the ascorbate peroxidase enzyme of *Leishmania*. MD simulation studies showed that ML-240 is an inhibitor of APX and greatly reduced the health and growth of the promastigotes. Similar to miltefosine, ML-240 induced a concentration-dependent reduction in the viability of promastigotes. In addition, ML-240 was also potent in restricting the long-term survival and growth of the parasites. ML-240 significantly prevents the replication of amastigotes in RAW 264.7 cells and human monocyte-derived macrophages, in addition to downregulating the intensity of parasitism, defined as the phagocytic index. ML-240 treatment significantly alters the promastigote ultrastructure and downregulates the ATP levels. *L. donovani*-infected BALB/c mice treated with ML-240 significantly curtailed the splenic and liver parasite burden in a concentration-dependent manner. The efficiency of ML-240 treatment was on par with that of the reference drug miltefosine, which is widely used as a frontline drug candidate against kala azar (leishmaniasis). Based on these computational data and in vivo animal model studies, we proposed a new inhibitor, ZINC96021026 (ML-240), for evaluation as an antileishmanial agent (Figure 3).

### 5.4. Screening of Novel Inhibitors against Calcium ion Channels of Leishmania

In *Leishmania,* Ca^2+^ ions regulate several vital functions including attachment and entry inside macrophages. Human calcium channel inhibitors have a significant effect on the growth and survival of *Leishmania* in vitro. This suggests that the *L. donovani* Ca^2+^ ion channel (Ld-CC) is a potential drug target. Ld-CC regulates the Ca^2+^ ion concentration, which controls several functions including flagellar motion, mitochondrial oxidative metabolism, and entry inside host macrophages. Homology modeling of Ld-CC and docking studies of the ligand library of three datasets of 542 compounds of National Cancer Institute (NCI) diversity were performed for screening studies. ZINC17287336 and ZINC29590262 were selected as the best energy conformers, showing the highest binding affinity for the target (Ld-CC). The ligands interact with the residues in the active site of the Ld-CC pocket, indicating that the docked conformations are acceptable. Moreover, these two ligands have relatively higher binding affinity than nifedipine and verapamil, which are reported as inhibitors of calcium channels in humans with mild anti-leishmanial activity. ZINC29590262 showed better binding and affinity toward Ld-CC than the human voltage-dependent alpha-1C subunit of the L-type calcium channel. This ligand showed >40% binding affinity with Ld-CC compared with human-VDCC, suggesting leishmanicidal potential [198] (Figure 3). The role of a secondary messenger such as the Ca^2+^ ion regulates a wide range of cellular processes in all eukaryotic organisms [213]. The mitochondrion of the parasite (*Leishmania* spp.) constitutes 12% of the total volume, forming an electrochemical gradient to provide a driving force for Ca^2+^ entry [214]. Homeostatic disturbance in intracellular Ca^2+^ ions may lead to lethal morphological defects, leading to apoptosis and cell death [214]. Calcium channel blockers (CCBs) are a class of compounds that are used for hypertension and other heart ailments in humans. Targeting calcium channels could be an effective strategy for exploring new drug development strategies against *Leishmania.* The docking study indicates that the designed inhibitors interact with the active site residues inside the pocket of the channel, which could hinder the entry of Ca^2+^ ions into the parasite and thereby jeopardize intracellular Ca^2+^ ion homeostasis and deter parasite survival in the host. Many compounds have attracted the attention of researchers and have undergone clinical trials with the aid of structure-based drug design (SBDD) [215].

### 5.5. Molecular and Cellular Aspects of Novel Drug Design

Widespread drug resistance against *Leishmania* severely impacts health care in areas where the disease is endemic. The need and urgency of new therapeutics against *Leishmania* need to involve the efficiency of leishmanicidal properties against newly developed clinical isolates and optimization of the compound for monitoring clinical trials. Chemotherapy is considered as the main treatment option against leishmaniasis, although it is plagued with ever-increasing drug resistance. The development of new therapeutics against *Leishmania* is a pressing need. A number of metabolic pathways are essential for parasite survival and are considered prospective drug targets. Enzyme targets for biochemical characterization and their usage for drug targeting have not been extensively explored. Biosynthetic pathways including sterol, glycolytic, DNA topoisomerase, redox metabolism, polyamine biosynthesis, folate, proteases, mitogen activated protein kinase, etc. have been investigated to find new drug targets against trypanosomatid parasites including *Leishmania* [216]. In addition to the above-mentioned pathways, several other pathways are also critical for parasite survival. Phosphoenol pyruvate carboxykinase, pyruvate phosphate dikinase, UDP-galactopyranose mutase, etc. are essential for parasite survival. Many such enzymes are parasite specific, indicating that they are not present in the mammalian (human) host and thus offering added advantages in drug design. Resistance mechanisms against the currently used drugs include alteration in drug reduction/activation, reduced uptake, and heightened efflux/sequestration of the active molecules. In addition, the amplification of genes and the enhanced activity of the repair mechanisms following drug-induced damage also play roles in the induction of resistance. Thus, new drugs with specific microbicidal properties could free and protect the body’s physiology and immune system from cycles of failure that always pose serious challenges in therapeutic success. The absence of any viable vaccine candidate or alternative therapy against protozoan parasitic disease makes it a more urgent need for the current situation.

UDP-galactopyranose mutase (UGM) is another target and has been explored as a possible platform for antileishmanial drug targets. β-Galf is absent in humans and present in many human pathogens such as *Mycobacterium tuberculosis*, *Leishmania* spp, *Trypanosoma* spp, and *Aspergillus fumigatus* (*Af*) as a major cell surface component [217,218]. UGM is accountable for the virulence of these pathogens [219]. β-Galf biosynthesis requires UGM, and the enzyme is absent in the human host and is an attractive drug target against *Leishmania spp.* In *Aspergillus fumigatus*, UGM deletion leads to the loss of virulence and inhibits growth, resulting in defects in cell wall morphology [220,221]. UGM deletion increased the sensitivity of *A. fumigatus* toward antifungal drugs [220,221]. Targeted deletion of UGM from *L. major* resulted in the loss of establishment of infection in BALB/c mice [222]. Specific inhibitors against mycobacterial UGM such as synthetic aminothiazoles have been reported to inhibit microbial growth [217]. UGM inhibition of *Brugiamalayi* also significantly disrupts in vitro as well as in vivo antifilarial activity [223]. UDP binding to UGM induces conformational changes in the enzyme. This change causes movement of the loop, which resides opposite the substrate binding site, leading to closure of the active site and preventing diffusion of the substrate. This structural property may help in the inhibitor design against UGM [218].

In kinetoplastids including *Trypanosoma*, *Leishmania*, etc., catalase and glutathione (GSH) peroxidase are not present, and the absence of hydroperoxidase in these pathogens is based on the tryparedoxin pathway for regulating oxidative stress [224,225]. APX is a key constituent in the glutathione ascorbate cycle. Glutathione maintains a reducing atmosphere inside the cells and imparts a reduced state upon many cellular components [226]. A single copy of the APX gene of *L. major* plays a pivotal role in H_2_O_2_ detoxification, which is generated due to endogenous processes following external interferences that include the oxidative burst of parasite-infected macrophages or the drug metabolism of the parasite [227]. Ascorbate biosynthesis in kinetoplastids occurs in the glycosomal compartment. Treatment of catalase and peroxidase (heme-containing enzymes) with aminotriazole or sodium azide hinders the removal of H_2_O_2_ from amastigotes [225]. Overexpression of APX in *L. major* promastigotes enhanced the tolerance to oxidative stress-induced apoptosis. APX overexpression in the mitochondria of *L. major* (Lm-APX) protects the pathogen from oxidative stresses such as mitochondrial dysfunction, senescence in the cell, and alteration in cellular redox equilibrium [228]. APX gene knockdown in parasites subjected to continuous exposure to oxidative stress generates higher intracellular H_2_O_2_ content [228]. Ablation of the APX gene in *L. major* caused secondary effects in lipophosphoglycan (LPG) and metacyclogenesis with reference to gene expression, instigated by an alteration in the redox equilibrium of the parasites. The APX inhibitor ML-240 is a valosin-containing protein (VCP), and p97, a member of the AAA-ATPase protein inhibitor family, could be a potential candidate for leishmanicidal activity. The AAA-ATPase protein family is also involved in cellular functions including endoplasmic reticulum-associated degradation (ERAD), Golgi membrane reassembly, cell division, DNA repair, and autophagy [229,230]. Thus, targeting APX of *L. donovani* selectively alters the ultrastructure of the parasites and arrests ATP levels, unlike miltefosine, which also causes significant damage to the host cells.

The advantage of PPDK-specific inhibitors against *Leishmania* is their lack of toxicity toward human cells at very high concentrations, although they are less effective than miltefosine. PPDK was previously investigated as a logical candidate for the design and development of potential herbicides and new drugs [202,231]. An in silico study on PPDK was aimed at finding a brand-new inhibitor that is toxic to parasites but tolerant toward mammalian cells. PPDK fits well in the exploration of new therapeutics since it has different catalytic mechanisms for the glycolytic pathway.

**Figure 4:**
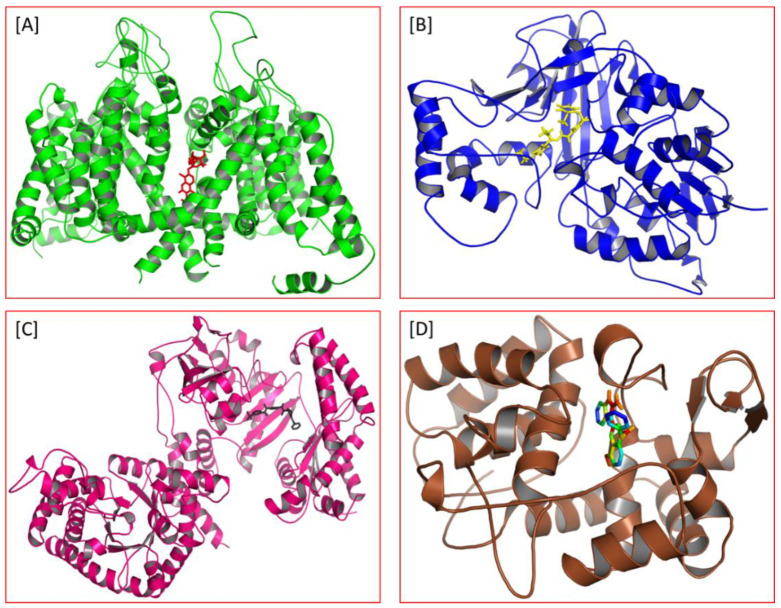
Docked complexes showing the interactions between the inhibitors and target proteins of *Leishmania*: *Ld*-CC ion channel is shown in green in the cartoon model and the inhibitor ZINC29590262 is in red in the stick model (**A**). The LdUGM, LdPPDK, and Ld-APX enzymes are shown in blue, pink, and brown in the cartoon model, respectively, while the corresponding inhibitors 6064500 (yellow), Z220582104 (black), and ML-240 (rainbow) are shown in the stick model inside the pocket of the respective proteins (**B**–**D**).

As a result, SBDD is now a useful instrument for the creation of new drugs in the field of medicine. To boost treatment effectiveness, avoid drug resistance, and administer less medication to prevent drug toxicity, computational approaches and software could be utilized to improve the prediction of innovative pharmaceuticals and synergistic pharmacological combinations. This article discusses the SBDD *Leishmania* parasite defense technique. Studies on molecular dynamics and docking point to a cellular and molecular interaction between the medication and a key parasite protein. This shows that a computational approach may help hasten the creation of new anti-trypanosomatid medications. Figure 4 shows the molecular interaction between the newly identified inhibitors with important enzymes and the calcium ion channel of *Leishmania*. We also documented the few data that have explored drugs based on SBDD against leishmaniasis (Table 5). The proteins were downloaded from RCSB PDB and presented in a cartoon model, while inhibitors were collected from the PubChem database (https://pubchem.ncbi.nlm.nih.gov/ (accessed on 2 Decemeber 2022)) and shown in a 2D model using the ChemSketch tool (https://www.acdlabs.com/ (accessed on 1 December 2022)). To find potential *Leishmania braziliensis* N-misristoyltransferase (LbNMT) inhibitors, a hierarchical virtual screening method based on the pharmacophore model, molecular docking, and molecular dynamics was used. PyMol is a structure visualization tool. We generated 3D images of proteins after downloading their structure from the RCSB-PDB database.

According to the docking, ZINC35426134 binding is thought to stabilize the enzyme. As a result, the chosen molecule may interact with the suggested target, which supports the SBDD strategy [232]. To find prospective lead compounds, in silico virtual screening of a natural product data collection containing 800 different chemical entities was conducted against the crystal structure of the *Leishmania infantum* trypanothione reductase (PDB ID: 2JK6). The different potential orientations that inhibitors can achieve in the active site of trypanothione reductase have been deduced from the foot printing of protein–inhibitor interactions [234]. This opportunity to find natural compounds with potential anti-leishmanial action has been made possible by this computational approach. Computational methods were also used to target certain *Leishmania* pathways and associated enzymes. One of the vital processes for *Leishmania* survival and pathogenicity is the glycolytic pathway. Intriguing interactions between various FDA-approved and antimalarial drugs and various glycolytic enzymes such as pyruvate kinase, triosephosphate isomerase, glucose-6-phosphate isomerase, glycerol-3-phosphate dehydrogenase, and glyceraldehyde-3-phosphate dehydrogenase have been discovered through molecular docking analysis. Another pathway study recommended important enzymes such as trypanothione reductase (TR), a key player in redox homeostasis, and tryparedoxin peroxidase. The active site of these enzymes exhibits an elective binding profile for ligands, according to the molecular docking data [75,233,235,236,237] (Figure 4). The interest in bioinformatics with respect to the cellular and molecular interactions has increased. The bioinformatics-based structure-based drug design (SBDD) approach has gained attention and has benefitted the search for novel drugs against leishmaniasis.

## 6. Future Perspectives

Understanding drug sensitivity and resistance is critical to safeguarding the efficacy of existing treatment options and to introduce new drugs in the future. Drug resistance is a critically important issue in leishmaniasis. Chemotherapy in leishmaniasis is plagued with drug resistance, which is evident from the less than desired clinical success rates, emergence of resistance, widespread toxicity, and/or cost of current drugs, suggesting an urgent need for new effective alternatives. Additionally, new effective preclinical studies in experimental animal models representing the various forms of the disease manifestations are needed. It is extremely important to study the appropriate animal models to test novel drug candidates. Preclinical evaluation should be agreed upon and compared with the works of other investigators. The adoption of combination therapy is important for extensive clinical tests of combination schemes to introduce quantitative and qualitative changes in therapy. The goal for new effective drug development needs result-oriented collaborative research in leishmaniasis, specifically in VL, due to its alarming record of fatality. The development of new, modern, cost-effective species-specific diagnostic methods needs to be explored for wide accessibility for cutaneous and visceral forms of the disease.

Proposed actions to select preclinical candidates for the treatment of VL.

1. Stronger need to find a broader range of active molecules, either new or repurposed, against *Leishmania*. Repurposed drugs need to be ‘true-and-tried’ with possible better outcomes in clinical management. New small molecules are desired as anti-leishmania drug candidates with reference to their effectiveness and low toxicity as well as their low market price.

2. Identification of multitarget drugs with a strong assurance of success. New drug discovery poses some challenges due to the complexity of having to validate action on the various targets. The application of computational chemistry is important for the initial screening and for application in multitarget quantitative structure analysis relationship analysis to predict the activity of a compound with a single model.

3. Validation of mean throughput systems (MHSs) and high throughput systems (HTSs) by means of a revision of the targets and parasite stages including the relevant parasitic stage (i.e., intracellular amastigotes) and validated molecular or pathway target. Avoidance of irrelevant targets by combining automatic and phenotypic screening is necessary.

4. Application of machine learning (ML) and artificial intelligence (AI) as novel approaches to overcome challenges, viz., the cost of developing new drugs, systemic toxicity, and evolving drug resistance with reference to the current regime of antileishmanial chemotherapeutics. Attention should be given to the growth of computer processing and the development of advanced algorithms. ML algorithms and AI can be instrumental in various applications of drug discovery and improve the current process and understanding of the cause and prevention of failure in clinical trials and regulatory approval.

5. ML and AI could screen millions of compounds to predict the optimal binding that potentially inhibits the function of parasites *Leishmania* or *Trypanosoma*, presenting proper PK/PD properties to enter clinical trials. DNDi and other PDPs sought to proceed on the scale required for drug discovery.

6. ML can be employed to address the drug resistance issue that has emerged in NTD pathologies. Protein transporters (P-gp, ABC transporter, etc.) play an important role in less than optimum uptake or enhanced efflux of the drug from the pathogen and initiate drug resistance. Computer-aided algorithms may identify the parasite-specific motifs of the responsible protein transporters in *Leishmania,* facilitate the allosteric modulation of drug transporters, and subsequently reduce parasite resistance.

7. Appropriate and high-content transparent evaluation of the safety and toxicity in suitable models is necessary. Evaluation of the selectivity index ex vivo (against amastigotes) and toxicity in vivo (maximum tolerated dose in a standard animal model) needs to be performed. Ethical requirements and scientific evidence should be implemented for in vivo preclinical trials performed in surrogate or advanced models that need supervision from specialists (e.g., veterinarians) with expertise in the pathophysiology of animal models.

8. Stringent evaluation of the effective concentration (EC) is needed. EC90 instead of EC50 is appropriate to reduce the number of potential hits to be tested for leishmanicidal potential in vitro and ex vivo. A combination of stringent EC90 and unbiased, transparent experimental animal studies should be adopted to reach further conclusions.

9. PK/PD characterization of new inhibitors/drugs including administration routes and the inclusion of standard animal models (e.g., mice, hamsters) and nonrodents (e.g., dogs) must be preceded by preliminary pharmacological characterization (e.g., snapshot method) to determine the major pharmacological parameters (AUC, availability, half-life, excretion rate, biodistribution of the molecule, etc.). The evaluation of new molecules for in vivo antileishmanial efficacy without mandatory scientific or ethical justification may be risky and potentially nonbeneficial. Collaboration between medicinal chemists and pharmacists, immunologists, and physiologists may improve the selection of a suitable molecule for optimal presentation.

10. Socioeconomical and anthropological issues cannot be ignored in drug discovery aspects of NTDs. Human-driven environmental occurrences including deforestation, climate change, and migration contribute greatly to the dissemination of diseases such as leishmaniasis including alterations in the sylvatic and domestic cycles of parasites. This also increases resistance events and the possibility of encountering exotic strains with unknown pathophysiology. The use of standard drugs such as miltefosine in combination with newly characterized parasite-specific exclusive drugs may be a pathfinder in this kind of scenario.

## 7. Conclusions

New drug discovery in leishmaniasis is rooted in trial-and-error strategies based purely on phenotypic screenings and occasional testing in animal models of the disease. Despite significant advancements in therapeutics, understanding of the more intricate molecular aspects of the disease including parasite biology and cellular and molecular mechanisms of host–parasite interactions still baffles the entire spectrum of health care professionals. This paradigm reflects many unknown issues regarding trypanosomatids, specifically their drug resistance and antigenic variation potential, which is likely to be related to the cellular and molecular aspects of the disease. With the advent of the genome project in early 2000, followed by the development of a wide array of computational software and databases, the exploration of new drugs against parasitic diseases including trypanosomatids has gained momentum. The role of pharmaceutical companies, not-for-profit organizations, and academic or research institutions have together brought scientific and technological developments in the areas of genomics, proteomics, and structural biology targeting NTDs including leishmaniasis. The contribution of data banks and virtual platforms viz. Sanger Institute’s GeneDB, TDR Targets Database (WHO), etc. have been made available and organize the data of the *Leishmania* species, emphasizing particular gene sequences and functions connecting diverse protein and small molecule libraries. The TDR Targets Database algorithm can generate privileged combinations of novel molecular targets and compounds for experimental evaluation. LmSmdB (Leishmania major and Schistosoma mansoni database) is a comprehensive database that uses computation to account for biological networks and regulatory pathways. It streamlines and simplifies the procedure for integrating the chemicals, genes, and protein structure in order to model a disease network and assist in the selection of a molecular target. Another database, LeishMicrosatDB (Leishmania Microsatellite Database), helps in the genome-wise mining and distribution of microsatellites throughout the parasite genome. The purpose of this is to give parasitologists a platform to comprehend the characterization, mapping, phylogeny, and evolutionary analysis of genomes. The database can assist scientists choose markers at specific intervals across the chromosomes, which can be useful for immunoinformatics investigations and diagnostics. These details produce a crucial understanding of parasite diversity and molecular machinery, which is a critical factor for developing broad-spectrum antileishmanial drugs. DeepMind created an artificial intelligence (AI)-based system called AlphaFold. Based on the arrangement of the amino acids in a protein, it predicts the three-dimensional structure of the protein. This tool is also gaining interest in target structure prediction and making contributions to drug design [238,239].

This information generates a key understanding of interspecies variability and molecular machinery in parasites, which is a critical factor for developing broad-spectrum antileishmanial drugs. The Drugs for Neglected Diseases initiative (DNDi) is another Lead Optimization Latin America (LOLA) consortium focused on preclinical assessment including the pharmacokinetic efficacy and safety of the designed drugs. Chemoinformatics, in addition to experimental evaluation including in vitro and in vivo animal model studies, could provide the structure–property and structure–activity relationships that could guide the design of optimized products.

## Figures and Tables

**Table 2:** Identification of novel antileishmanial drugs specific to biochemical pathways critical for the survival of *Leishmania donovani*.

Pathway	Drug Target	Drug Candidate	Mode of Action	Refs.
Sterol Biosynthesis Pathway	Squalene epoxidase	Spiro[indole-3,3′-pyrrolizidine]-2-one	DNA topoisomerase IB inhibitor.	[67,68]
	HMGR enzyme	Mevastatin	Hampers HMGR activity.	[69,70]
	Sterol alpha-14 demethylase	Avodart	Induces ROS and causes apoptosis in the parasite.	[71]
	HMGR enzyme	Glycyrrhizic acid	Inhibits HMGR enzyme.	[72]
Purine Salvage Pathway	mRNA translation	5-fluorouracil 4-thiouracil	Binds to RNA and blocks cell growth.	[73,74]
Glycolytic Pathway	GAPDH	Artesunate	Inhibits the parasites’ glycolytic enzymes GPDH.	[75,76]
		Quinine		[75]
		Mefloquine		[75]
Folate Biosynthesis Pathway	DHFR	Methotrexate (MTX, 1)	Inhibits DHFR.	[77]
		Cycloguanil		[77]
		Trimethoprim (TMP, 2)		[77,78]
		ZINC57774418 (Z18)	Inhibits DHFR activity.	[79]
		ZINC69844431 (Z31)		[79]
		ZINC71746025 (Z25)		[79]
		D11596 (DB96)		[79]
		3,4-dihydropyrimidine-2-one		[80]
		5-(3,5-dimethoxybenzyl) pyrimidine-2,4-diamine		[80]
	DHFR and PTR1	2-(4-((2,4-dichlorobenzyl)oxy)phenyl)-1H-benzo[d]imidazole	DHFR-TS/PTR1 inhibitors.	[81]
		2-(4-((2,4-dichlorobenzyl)oxy)phenyl)-1H-benzo[d]imidazole-1H-benzo[d]oxazole		[81]
Trypanothione Pathway	TR	Trichloro [1,2-ethanediolato-O,O’]-tellurate (AS101)	Induces ROS-mediated apoptosis by binding to TR cysteine residues.	[82]
		β-sitosterol CCL	Inhibit TR activity.	[83]
Hypusine Pathway	Spermidine synthase	Hypericin	ROS and spermidine reduction.	[84,85]

**Table 3:** The major computational tools used in the in silico analysis for the identification of novel leishmanicidal compounds derived from the available databases.

S. No	Resources	Descriptions	Weblink	Ref.
1.	TriTrypDB	For *Leishmania* and *Trypanosoma,* an integrated genomic and functional genomic resource is available.	http://tritrypdb.org (accessed on 8 December 2022)	[166]
2.	LeishCyc	*L. major* biochemical pathway database.	http://biocyc.org/LEISH/organism-summary?object¼LEISH (accessed on 7 December 2022)	[167]
3.	*L. amazonensis* genome DB	The genome of L. amazonensis has been sequenced and annotated.	http://bioinfo08.ibi.unicamp.br/leishmania (accessed on 6 December 2022)	[168]
4.	GeneDB (Kinetoplastid Protozoa section)	Annotations and sequences of 5 *Leishmania* species were curated.	http://www.genedb.or (accessed on 8 December 2022)	[169]
5.	EuPathDB	For eukaryotic pathogens, there is a pathogen genomics resource.	http://eupathdb.org (accessed on 9 December 2022)	[170]
6.	LmSmdB	Regulatory pathways and biological networks of *L. major*.	http://www.nccs.res.in/LmSmdb (accessed on 9 December 2022)	[171]
7.	LeishMicrosatDB	Repeat sequences from six *Leishmania* species are included in a database.	http://biomedinformri.com/leishmicrosat (accessed on 11 December 2022)	[172]
8.	TrypsNetDB	Protein interactions and annotations for trypanosomatid parasites that have been experimentally verified as well as predicted.	http://trypsNetDB.org (accessed on 5 December 2022)	[173]
9.	LeishDB	Noncoding RNAs and coding gene reannotation in *L. braziliensis.*	http://www.leishdb.com (accessed on 7 December 2022)	[174]
10.	List of putative anti-leishmanials	Lead compounds and drug targets with predicted antileishmanial activity.	https://www.ncbi.nlm.nih.gov/pmc/articles/PMC4247209/(accessed on 6 December 2022)	[175]
11.	*L. major* metabolic network	Genome-scale metabolic network of *Leishmania major* (iAC560).	https://www.ebi.ac.uk/biomodels/MODEL1507180059 (accessed on 10 December 2022)	[176]

**Table 4:** List of novel drugs explored by chemoinformatics against *Leishmania donovani*.

Drug	Structure	Pathway	Target Protein	Mode of Action	Ref.
Z220582104	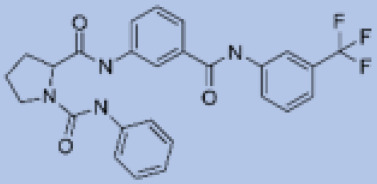	Glucose synthesis and alanine influx	Pyruvate phosphate dikinase (PPDK)	Inhibits the pyruvate phosphate dikinase enzyme that helps in Alanine entry Mannogen synthesis from lactate ATP formation	[179]
CID 6064500	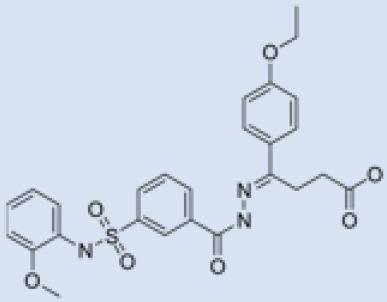	Β-Galf synthesis Role in pathogenesis	UDP-galactopyranose mutase (UGM)	Inhibits the UDP-galactopyranose mutase (UGM) enzyme It catalyzes the UDP galacto pyranose (UDP-Galp) to UDP-galacto furanose (UDP-Galf) UGM helps in plays an important role in the pathogenesis of the parasite	[197]
ZINC96021026	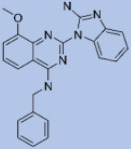	Tryparedoxin	Ascorbate peroxidase (APX)	Inhibits the ascorbate peroxidase enzyme activity It is a redox enzyme (tryparedoxin pathway) It helps in H_2_O_2_ removal or its detoxification	[180]
ZINC29590262	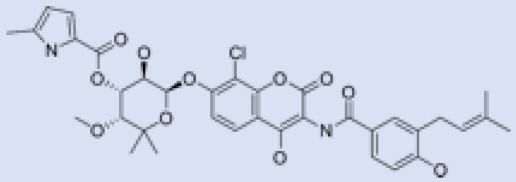	Ca^2+^ related pathways	Calcium channel	Inhibits calcium channel that hampers flagellar motion Inhibit mitochondrial function and oxidative metabolism Affects the Ca^2+^ pathways	[198]

**Table 5:** List of proteins and drugs explored by the structure-based drug design against different *Leishmania* species. The cartoon model of the protein structure was generated using the PyMol tool and shown in different colors. The 2D structures of the compounds were generated by ChemSketch 3D.

S. No	*Leishmania* Spp.	Target Proteins	Structure of the Protein	Compound	Ref.
1.	*Leishmania major*	N-myristoyl transferase (PDBID: 5A27)	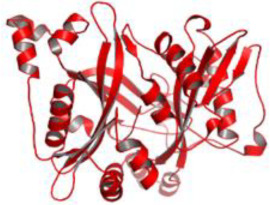	ZINC35426134 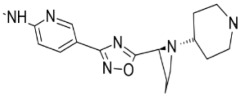	[232]
2.	*Leishmania major*	Tryparedoxin peroxidase (PDB ID: 3TUE)	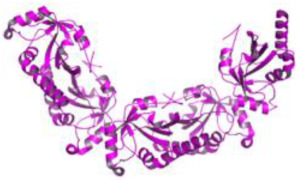	Taxifolin 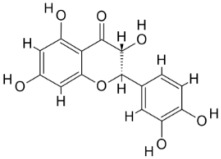	[233]
				Quercetin 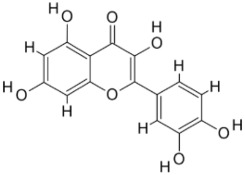	
3.	*Leishmania infantum*	Trypanothione reductase (PDB ID: 2JK6)	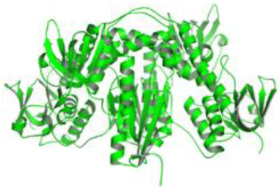	Beta-Amyrin Acetate 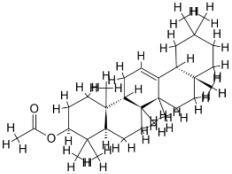	[234]
				Ginkgetin 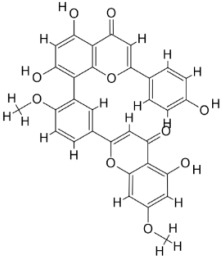	
				Fucostanol 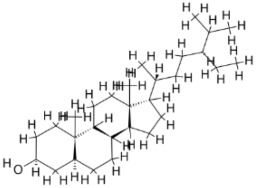	
				Lunarine 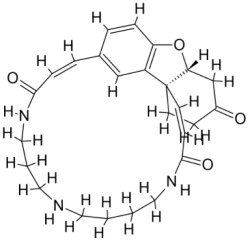	
4.	*Leishmania infantum*	Trypanothione reductase (PDB ID: 5EBK)	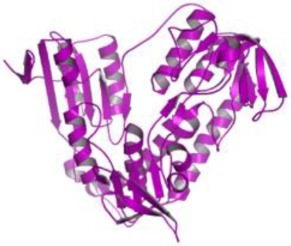	Masticadienonic acid 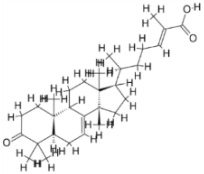	[235]
5.	*Leishmania infantum*	Trypanothione reductase (PDB ID: 2JK6)	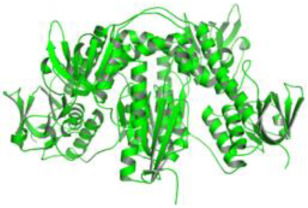	Epigallocatechin Gallate 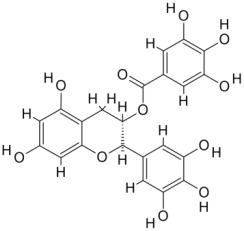 (EGCG)	[236]
6.	*Leishmania mexicana*	Pyruvate kinase (PDB ID: 3PP7)	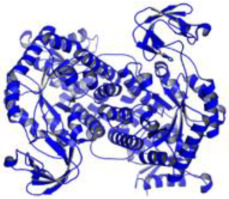	Irinotecan	[237]
				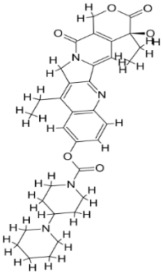	
				Coniveptan	
				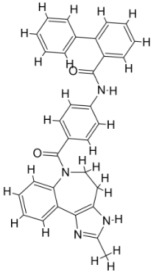	
				Valstar	
				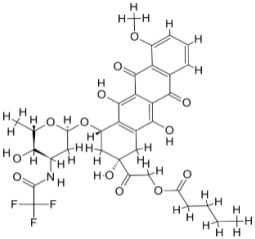	
				Nilotinib	
				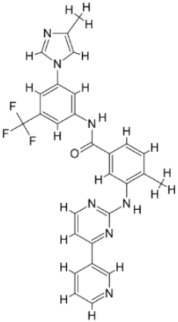	
				Netupitant	
				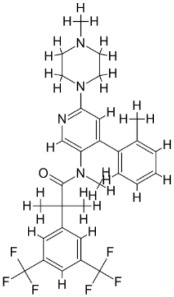	
				Lomitapide	
				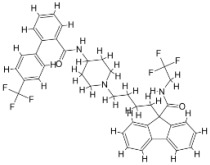	
				Trametinib	
				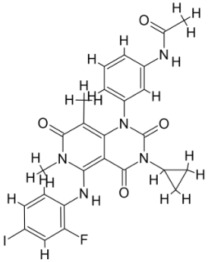	
				Naldemedine	
				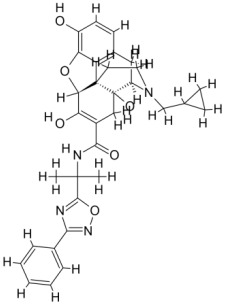	
				Vumon	
				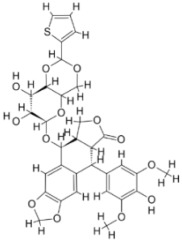	
				Eltrombopag	
				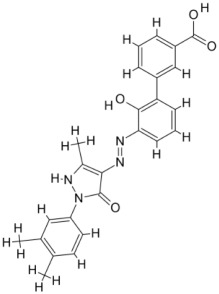	
7.	*Leishmania mexicana*	Glucose-6-phosphate isomerase (PDB ID: 1T10)	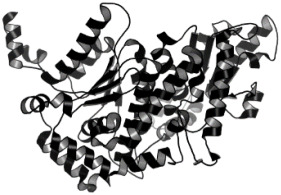	Artesunate	[75]
				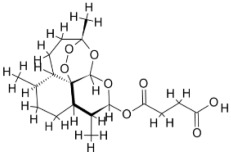	
				Quinine	
				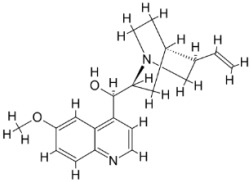	
	*Leishmania mexicana*	Triosephosphate isomerase (PDB ID: 2Y63)	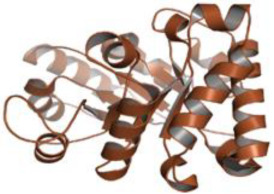	Mefloquine	
	*Leishmania mexicana*	Glycerol-3-phosphate dehydrogenase (PDB ID: 1M67)	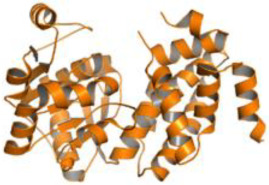		
	*Leishmania mexicana*	Glyceraldehyde-3-phosphate dehydrogenase (PDB ID: 1I33)	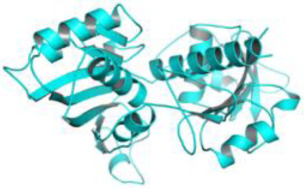	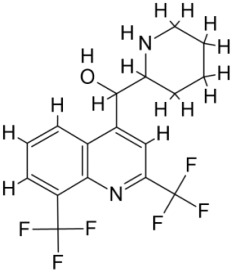	
	*Leishmania mexicana*	Pyruvate kinase (PDB ID: 3PP7)	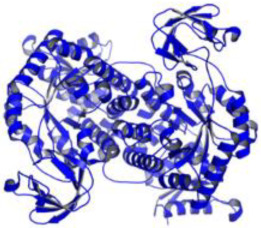		

## Data Availability

Not applicable.

## Abbreviations

APX	Ascorbate peroxidase
CL	Cutaneous leishmaniasis
DB	Database
DHFR	Dihydrofolate reductase
DHFR-TS	Bifunctional dihydrofolate reductase-thymidylate synthase
FDA	Food and Drug Administration
GAPDH	glyceraldehyde 3-phosphate dehydrogenase
H_2_O_2_	Hydrogen peroxide
HIV	human immunodeficiency virus
HMGR	HMG-CoA reductase
mRNA	Messenger RNA
PDB	Protein Data Bank
PKDL	Post Kala-azar dermal leishmaniasis
PLGA	poly lactic acid (PLA) and poly glycolic acid (PGA)
PRP1	Proline-rich protein 1
PTR1	Pteridine reductase 1
ROS	Reactive oxygen species
Top1	DNA topoisomerase I
TR	Trypanothione reductase
UDP	Uridine diphosphate
UGM	UDP-galactopyranose mutase
VL	Visceral leishmaniasis
